# Development of a Gravid Trap for Collecting Live Malaria Vectors *Anopheles gambiae s.l.*

**DOI:** 10.1371/journal.pone.0068948

**Published:** 2013-07-05

**Authors:** Sisay Dugassa, Jenny M. Lindh, Florence Oyieke, Wolfgang R. Mukabana, Steven W. Lindsay, Ulrike Fillinger

**Affiliations:** 1 International Centre of Insect Physiology and Ecology (icipe)-Thomas Odhiambo Campus, Mbita, Kenya,; 2 School of Biological Sciences, University of Nairobi, Nairobi, Kenya; 3 Royal Institute of Technology, Stockholm, Sweden; 4 School of Biological and Biomedical Sciences, Durham University, Durham, United Kingdom; 5 Department of Disease Control, London School of Hygiene and Tropical Medicine, London, United Kingdom; University of Crete, Greece

## Abstract

**Background:**

Effective malaria vector control targeting indoor host-seeking mosquitoes has resulted in fewer vectors entering houses in many areas of sub-Saharan Africa, with the proportion of vectors outdoors becoming more important in the transmission of this disease. This study aimed to develop a gravid trap for the outdoor collection of the malaria vector *Anopheles gambiae* s.l. based on evaluation and modification of commercially available gravid traps.

**Methods:**

Experiments were implemented in an 80 m^2^ semi-field system where 200 gravid *Anopheles gambiae s.s.* were released nightly. The efficacy of the Box, CDC and Frommer updraft gravid traps was compared. The Box gravid trap was tested to determine if the presence of the trap over water and the trap’s sound affected catch size. Mosquitoes approaching the treatment were evaluated using electrocuting nets or detergents added to the water in the trap. Based on the results, a new gravid trap (OviART trap) that provided an open, unobstructed oviposition site was developed and evaluated.

**Results:**

Box and CDC gravid traps collected similar numbers (relative rate (RR) 0.8, 95% confidence interval (CI) 0.6–1.2; p = 0.284), whereas the Frommer trap caught 70% fewer mosquitoes (RR 0.3, 95% CI 0.2–0.5; p < 0.001). The number of mosquitoes approaching the Box trap was significantly reduced when the trap was positioned over a water-filled basin compared to an open pond (RR 0.7 95% CI 0.6–0.7; p < 0.001). This effect was not due to the sound of the trap. Catch size increased by 60% (RR 1.6, 1.2–2.2; p = 0.001) with the new OviART trap.

**Conclusion:**

Gravid *An*. *Gambiae* s.s. females were visually deterred by the presence of the trapping device directly over the oviposition medium. Based on these investigations, an effective gravid trap was developed that provides open landing space for egg-laying *Anopheles* .

## Introduction

Vector control plays a central role in the prevention of malaria [1–5]. Monitoring vector populations and assessment of disease risk are among the key elements of vector management strategies [6–9]. So far various tools have been developed and utilized for sampling mosquito vectors and the pathogens they transmit [10–13]. In sub-Saharan Africa (SSA) the most commonly used sampling methods for malaria vectors are human landing catches, CDC light traps, and pyrethrum spray collections which are excellent for sampling host-seeking and indoor resting mosquitoes [14–16]. Effective vector control targeting host-seeking and resting mosquitoes indoors has resulted in a reduction in the number of mosquitoes entering and resting in houses [11,17–24] rendering some of these tools less effective for monitoring potential vector populations [25].

Collecting malaria vectors outdoors becomes increasingly important as surveillance tools; and effective traps might even be used for control purposes [26]. To date outdoor vector collections target either resting populations with pit trap [27,28], pot traps [13], resting boxes [29] and aspirator collections or host-seeking mosquitoes with animal-baited traps [13,30,31], human baited tent traps [32] or human landing catches [14,16] and, more recently, odour-baited MM-X traps [33]. In general, outdoor sampling is far more challenging than indoor sampling since the outdoor vector population is more dispersed over the landscape. Resting catches often underestimate actual vector densities [19] and odour-baited traps only target a proportion of the host-seeking population (either attracted to animals or to humans). Furthermore, animal or human-baited traps are complicated to organise and are inappropriate for using on a large scale.

Gravid traps would provide an important and novel sampling tool for sampling both endophilic and exophilic vector populations in search of an oviposition site, a grossly understudied phase in the life cycle of *Anopheles* mosquitoes. Gravid mosquito traps are designed to catch gravid females in search of an aquatic habitat. Such traps are used routinely for the surveillance of *Culex* and *Aedes* vectors [34,35] but not for gravid *Anopheles gambiae* s.l., the major malaria vector in SSA, Harris and collegues [36] have recently developed the first sampling tool for this species consisting of a transparent acetate sheet coated with insect glue that is placed on the water surface on the edge of a natural habitat. Their pilot study in an area of high vector densities showed that gravid culicines and anophelines in search of an oviposition site landed and got stuck on the transparencies [36]. Further studies need to confirm the effectiveness of this sticky transparency in areas of low mosquito and habitat densities. Here we were interested to evaluate suction traps that sample mosquitoes alive, that provide the opportunity to be moved around and do not depend on the presence of natural habitats. The first was developed by Reiter in 1983 [37] to collect gravid *Culex* and *Aedes* mosquitoes for West Nile virus isolation [34,38,39] and many gravid mosquito traps have been developed and modified since then [38,40,41]. Among these, the CDC gravid trap Model 1712, commonly referred to as CDC gravid trap, the CDC gravid trap Model 1719 commonly referred to as Frommer updraft gravid trap and the Box gravid trap (also referred to as Reiter-Cummings gravid trap) [38] are commercially available. These widely used traps are suction traps for the collection of live, gravid culicines [38,40]. However, to our knowledge, none of these have been purposely evaluated for collecting *An. gambiae* s.l. nor anophelines in general.

Here we aimed to investigate the factors that impact on the catching efficiency of these commercially available traps. Based on the results a new prototype gravid trap for the collection of malaria vectors was developed.

## Materials and Methods

### Study area

The study was carried out using a semi-field system [42] located at the International Centre of Insect Physiology and Ecology, Thomas Odhiambo Campus (icipe-TOC), Mbita, on the shores of Lake Victoria, Kenya (0^o^ 26’ 06.19’’S, 34^o^ 12’ 53.13’’ E; altitude 1,137m above sea level). This area is characterised by a consistent tropical climate with an average minimum temperature of 18°C and an average maximum temperature of 28°C (based on data from icipe-TOC meteorological station for 2010-2012). The area experiences two rainy seasons, the long rainy season between March and June and the short rainy season between October and December. The average annual rainfall for 2010-2012 was 1,436mm.

### The semi-field system and study design

**Figure 1:**
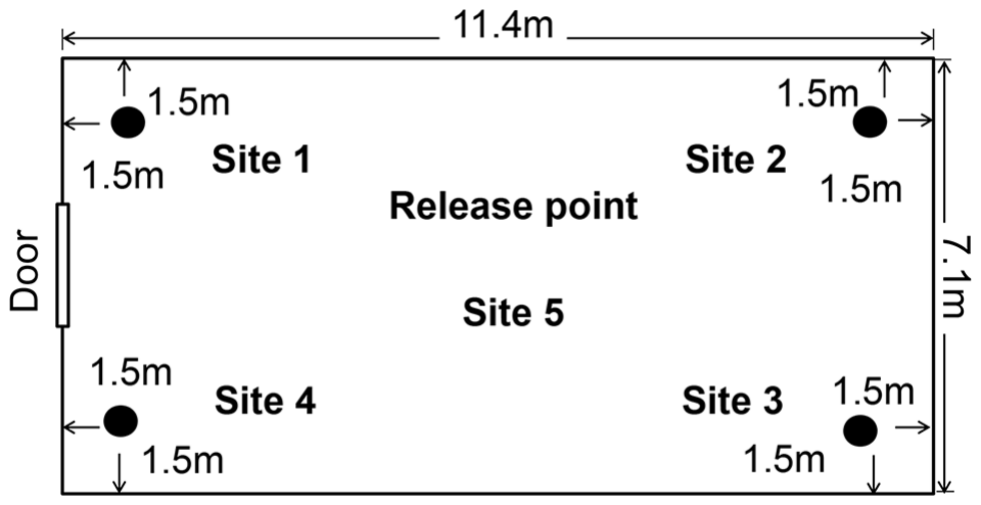
Schematic drawing of the dimension of the semi-field system, the treatment sites (1–4) and release point (site5).

The semi-field system was a screened building 7.1 m wide, 11.4 m long and 2.8 m high at the wall and 4.0 m high at the highest point of the roof. The two opposite shorter (7.1m wide) walls and roof were made of glass and the two long walls were screened with black fibre glass netting gauze (1.7 × 1.5 mm). The floor was covered with sand to a depth of 30 cm so that artificial aquatic habitats could be dug into the ground to simulate a natural larval habitat for the *An. gambiae s.s.* mosquitoes [43]. To increase the relative humidity inside the semi-field system to 60-70% the sand floor was watered from 15:00-16:00 h prior to the experiment. Care was taken to ensure that no pooling of water occurred on the floor and that the upper layer of sand was dry when the mosquitoes were released. Treatments were positioned in the corners of the semi-field system at a distance of 1.5 m from the two adjacent walls (site 1-4) and mosquitoes released from the centre (site 5; Figure 1). Experiments were conducted using randomized complete block designs (RCBD) and replicated for 12 nights. The number of replications was based on sample size considerations for comparing proportions of clustered data [44]. The preliminary data for this were generated by setting two identical artificial ponds in opposite corners of the semi-field system. The water in the ponds was treated with detergent as described in detail by Dugassa and colleagues [43]. Two hundred visually presumed gravid females (see details below) were released in the evening and the number of mosquitoes drowned in each pond was counted the next morning. This was done for 12 nights. The results showed that when presented with an identical treatment the gravid females approached both ponds in an equal proportion (p1=0.5). The variability of the nightly catches was used to calculate the coefficient of variation (ratio of standard deviation/mean) which was 0.26. At this ratio, replication of the experiment over 12 nights, assuming 100 responders out of 200 released mosquitoes per night, had 80% power to detect an increase or decrease in the catch rate of 20% (p2=0.7) at the 5% level of significance. This level of accuracy was appropriate for developing new traps for gravid *An. gambiae s.s.*. since we were looking to develop traps that were markedly better at collecting gravid mosquitoes than established traps, thus were interested in designs that increased trapping by at least 20%.

### Mosquitoes

Insectary-reared *An. gambiae* s.s. mosquitoes were used throughout. Gravid mosquitoes were prepared as follows; 300 female and 300 male mosquitoes, two to three days old, were selected from the rearing cages at midday and kept in 30 cm × 30 cm × 30 cm netting cages at ambient conditions of 25-28°C and a relative humidity of 68-75%. Water saturated cotton towels (50 x 25cm) were folded and placed over the cages to avoid mosquito desiccation. Mosquitoes were starved of sugar for seven hours prior to blood feeding and allowed to feed on a human arm for 15 minutes at 19.00h on the same day. The same procedure was repeated 24 hours later. After the first blood meal unfed female mosquitoes were removed from the cages. After feeding mosquitoes were provided with 6% glucose solution *ad libitum*. Fed female mosquitoes were kept together with males for two days after the second blood meal before they were utilised in an experiment (i.e. females 4-5 days after first blood meal). At 16.30 h at the day of experiment 200 females with an enlarged, pale white abdomen were collected from the holding cage and visually presumed gravid (henceforth referred to as gravid females). A small proportion of these mosquitoes might not have been gravid because most females need two blood meals to reach full gravidity [45] and some never reach full gravidity even after three feeds [46]. Whilst we provided two meals we cannot guarantee that two meals were taken by all females.

### Gravid Traps

**Figure 2:**
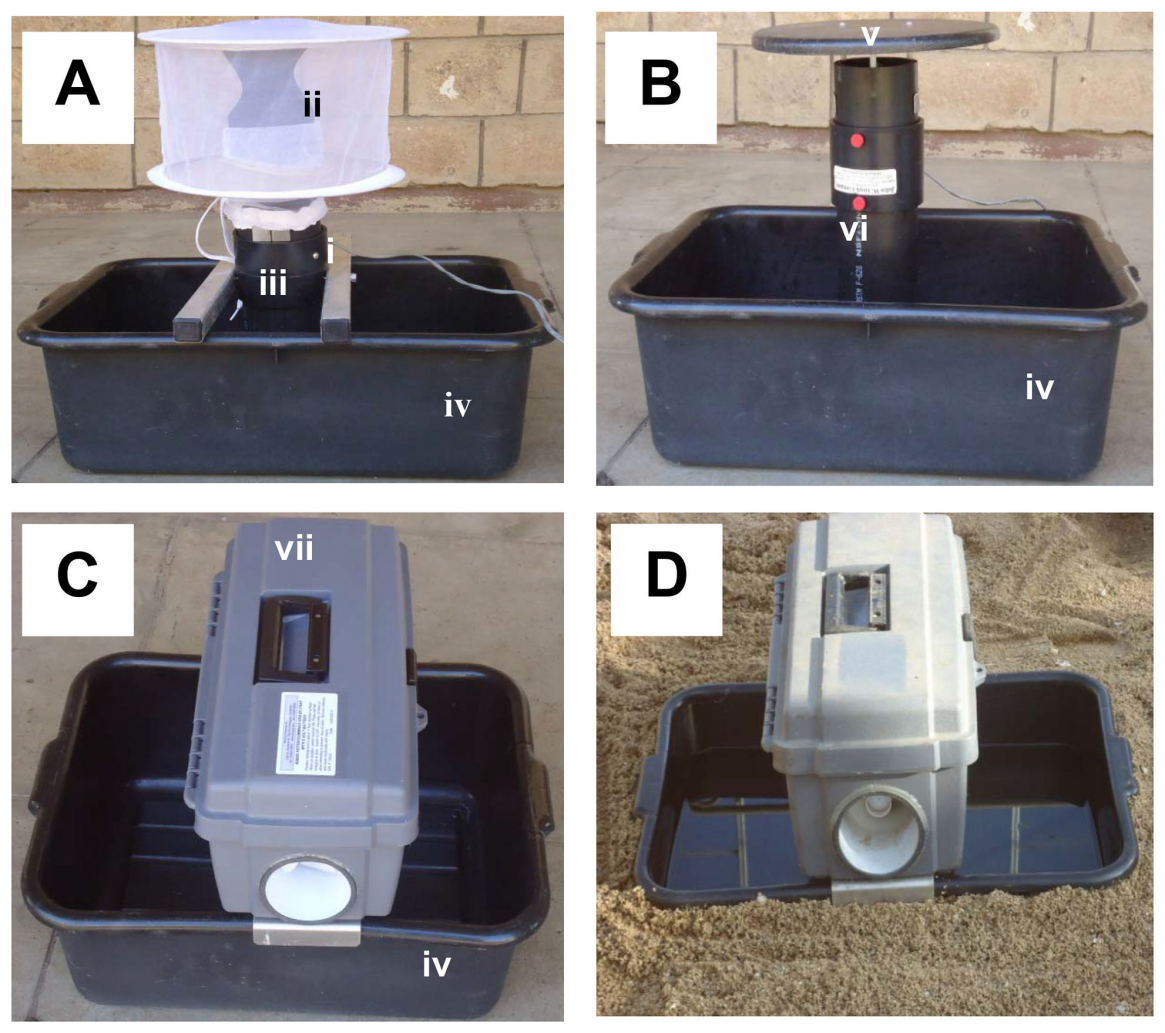
Set up of three gravid traps. (A) CDC gravid trap, (B) Frommer updraft gravid trap; (C) Box gravid trap. i) aluminium supports, ii) collection bag, iii) upright tube, iv) pan, v) rain shield, vi) base stand, vii) horizontal exhaust tube, viii) anti-spread bar; (D) Box gravid trap lowered into ground in semi-field system.

The Box gravid trap (BioQuip, Rancho Dominguez, CA), CDC gravid trap (John W. Hock Company, Gainesville, FL)) and Frommer updraft gravid trap (John W. Hock Company, Gainesville, FL) were used in this study. These traps attract egg-laying females to a water-based oviposition medium added to bowls below the trapping device. The bowls’ size varied slightly between different trap models. According to the manufacturers’ recommendations oviposition medium was filled in the bowls to a level of about 3 cm below the opening of the intake ducts. This is equivalent to 8 L of oviposition medium in the Box gravid trap, 6 L in the CDC and 5 L in the Frommer trap. All traps operate by drawing air from the surface of the bowls and distributing any volatile chemicals associated with the oviposition medium, including water vapour, to the surrounding of the traps. Depending on the design of the trap and the location of the air intake duct, the air plume varies amongst the traps [40,47]. In all traps mosquitoes are sucked into a collection chamber while they evaluate the potential oviposition site and prepare to lay eggs. The collection chambers are found on top of a suction tube in the Box and Frommer updraft gravid traps to avoid exposure of mosquitoes to the aspiration fan. However, the collection chamber of the CDC gravid trap is placed above the aspiration fan [38,40] so that some mosquitoes are damaged by the rotating fan [48,49]. The water basins of the traps are usually placed on the ground for collection of *Aedes* and *Culex* mosquitoes (Figure 2A, B, C) which will readily lay eggs in containers. However, *An. gambiae* s.l. usually prefer natural habitats so here we aimed to reduce the container impression by sinking the bowls in to the ground for all experiments, where we refer to the water-filled bowls as ponds (Figure 2D).

Figure 2 shows the three traps. The Box gravid trap was set up by fitting the anti-spread bars that are found under the horizontal exhaust tube to the black bowl (16.5 L volume, 44 cm long, 34 cm wide and 12 cm deep). The black conducting duct with the large ‘O’ ring around it was placed into the hole at the bottom of the case. The wire screen of the collecting chamber was placed on the outside of the collecting duct with the intake screen facing the exhaust tube. The CDC gravid trap was set up by placing the aluminium supports of the trap on the rim of the pan (24 L volume, 44 cm long, 34 cm wide and 17 cm deep) and slipping the collection bag over the upright tube. The sleeve was slipped downwards towards the aluminium supports until the bottom of the bag rested on the top end of the trap. Setting up the Frommer updraft gravid trap involved fitting the rain shield with aspiration fan to the trap and setting it into the base stand. The parts were attached tightly. The base stand was placed in the black pan (24 L volume, 44 m long, 34 cm wide and 17 cm deep) so that the feet rested inside the tray.

For all experiments piped non-chlorinated water from Lake Victoria was filtered through a sand-charcoal filter and used in the containers of the traps as oviposition medium. All the gravid traps were operated by a fully charged 6 volt 12 Ah battery (Universal battery UB6120). Experiments were started at 17.30 h by releasing 200 gravid mosquitoes at the centre (site 5) of the semi-field system and stopped at 8.00 h the following day. Any live mosquitoes remaining in the semi-field system were removed and killed. The collection chambers from the traps were kept in a freezer for 30 minutes to kill the mosquitoes.

### Trap comparison

In the first experiment the trapping efficacy of the Box gravid trap, CDC and Frommer updraft gravid trap was compared. In addition to the three traps an open pond made of a similar bowl but without a trapping device was set up in the semi-field system. The open pond was positioned in the same site each night (site 4). Traps were rotated over the 12 nights between sites 1, 2 and 3. The purpose of the open pond was to serve as a reference to compare mosquito responses from night to night and to compare the relative attractiveness of a ‘natural’ water body with ones that had gravid traps. The number of mosquitoes trapped in the collection chambers of the traps and the number of eggs laid in each pond was recorded nightly. To count the number of eggs the water from the bowls was filtered through a filter paper (Fisherbrand, QL 125) using a water suction vacuum pump. The bowls were rinsed with additional water and white filter paper passed slowly along the edges of the bowls to detect any more eggs that might have remained after rinsing.

### Trapping efficacy of the Box gravid trap

After the first experiment the Box gravid trap was chosen for further evaluation to investigate factors that might affect catch size. Experiments were designed to assess if the position of the Box trap on top of the pond or the sound of the fan affected the number of gravid females that approach the trap. In the following experiments, 8L filtered-lake water was used to prepare the ponds.

**Figure 3:**
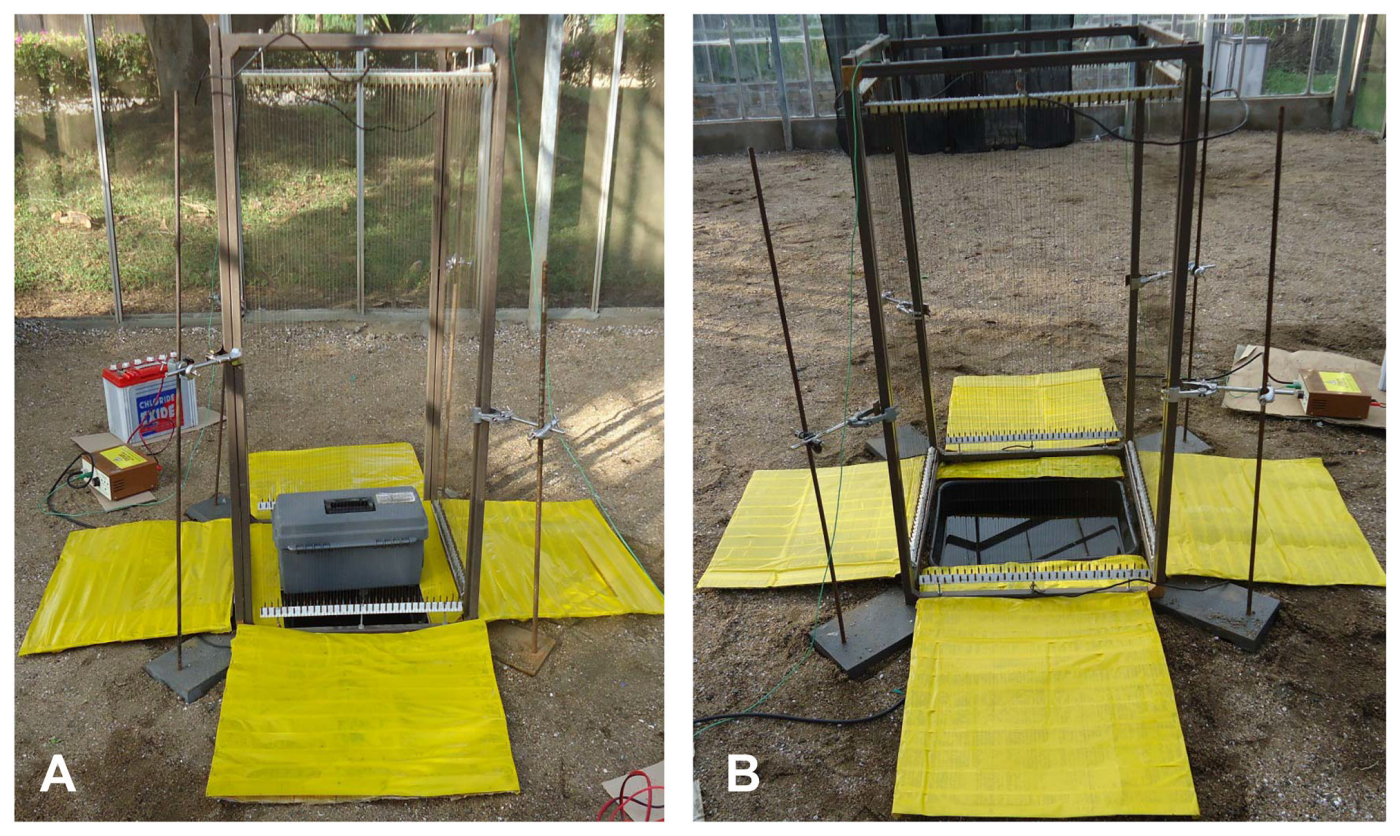
Box gravid trap (A) and open pond (B) surrounded by a square of electrocuting nets.

The first experiment had a fully-functional Box gravid trap in one corner of the semi-field system (Figure 3A) and an open pond (pond alone without trapping device) in the opposite corner (Figure 3B). In the second experiment the fan of the Box gravid trap was switched off to assess if the sound of the trap affected the number of gravid *Anopheles* mosquitoes approaching the pond. Here we compared a non-functional Box trap with an open pond in the opposite corner of the semi-field system. In both experiments treatments were rotated between all sites (site 1-4). To analyse the orientation of gravid females towards either of the ponds they were surrounded by a complete square of electrocuting nets (Figure 3). The adjacent wires of the nets were powered by a 12 V 50 Ah lead acid battery (Chloride Exide Ltd, Kenya) via a spark box (Alan Cullis, South Africa) adjusted to a low spark energy setting that did not produce any sound or spark but killed the mosquitoes that touched the net while approaching the pond. A detailed description of the electrocuting nets and spark box specifications can be found in a recent publication [43]. Yellow sticky films mounted on strips of cardboard served as collection boards (Figure 3). These were placed under each net outside the closed square (50 x 60 cm) and in the gaps between the two longer sides of the bowls and the net (53 x 7 cm). The number of mosquitoes approaching either pond was estimated by counting the number on the net and on the collection boards.

### Development of a new gravid trap (OviART gravid trap)

Results from previous experiments indicated that the presence of a Box on top of a pond affected the number of gravid females approaching this pond. Therefore we moved the Box trap from directly above the water of the pond to one side of the pond on the sand. The Box trap was positioned 50 cm from the edge of a pond and compared with an open artificial pond. The trap was not switched on as the aim was to test if the removal of the Box from the surface of the pond to the side would improve mosquitoes’ approach to the pond or if the presence of a box close to the pond would also deter mosquitoes. To quantify the number of mosquitoes visiting the two ponds 200 ml detergent (Teepol Industries LTD, Nairobi) was added to the water of each pond [43].

**Figure 4:**
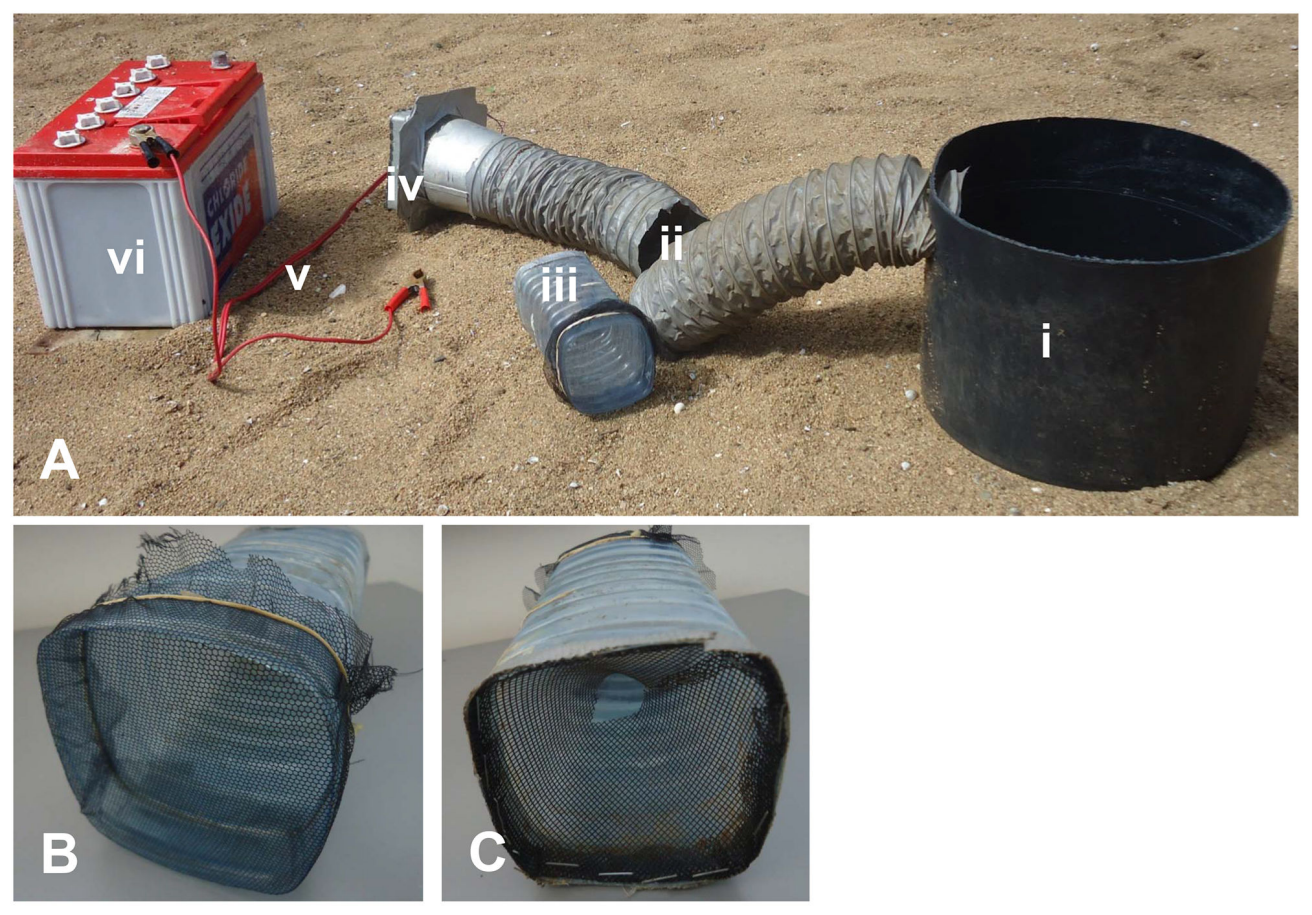
Ovi-ART gravid trap prototype. (A) Trap parts: i) round black bucket, ii) suction tube prepared from collapsible plastic pipe, iii) collection chamber prepared from plastic bottle and fiber glass netting gauze, iv) fan (12V, 0.38A), v) electric cable, vi) 12V battery. (B) Collection chamber backside towards fan. (C) Collection chamber entry funnel.

Based on our findings from the above experiments we constructed a new prototype gravid trap (‘OviART gravid trap’ named after the research project funding this work on **O**viposition of *Anopheles gambiae*: **A**ttractants, **R**esidual larvicides and **T**raps) where the collection chamber and fan were positioned on one side of the pond (Figure 4). A black round bucket (20 cm high and 30 cm in diameter) filled with 8 L of filtered-lake water served as oviposition site. An oval slit (13 cm wide and 5 cm high) was cut 5 cm below the lip of the bucket into which a collapsible pipe (30 cm long and 10.2 cm in diameter) was inserted. This pipe was connected to a collection chamber made out of a water plastic bottle as described below. At the end of the collection chamber another 30 cm collapsible pipe and a fan of 12 V and 0.38 Ah current output (as opposed to 6 V and 0.1 Ah of the Box gravid trap) was fixed to create strong air suction. A strong suction of air from the entire water surface was needed to compensate for the reduction in airflow as a result of moving the suction point from above the pond to the side. The fan sucked air into a collection chamber (20 cm long and 10 cm in diameter) which was prepared from a plastic water bottle of 1 L volume and black fiber glass netting gauze (1.7 mm × 1.5 mm mesh size). A piece of netting gauze (15 cmx15 cm) was cut and prepared into a conical funnel with a 2.5 cm wide hole at the narrower end and 10 cm at the wider end. It was then fixed at the inlet side of the water bottle with the narrower opening of the funnel positioned inside the bottle. This narrow inlet minimized risk of escape of mosquitoes even when the power stopped due to battery failure. Another piece of the gauze (18 cm × 18 cm) was cut and tied to the opposite side of the bottle towards the fan (Figure 4).

**Figure 5:**
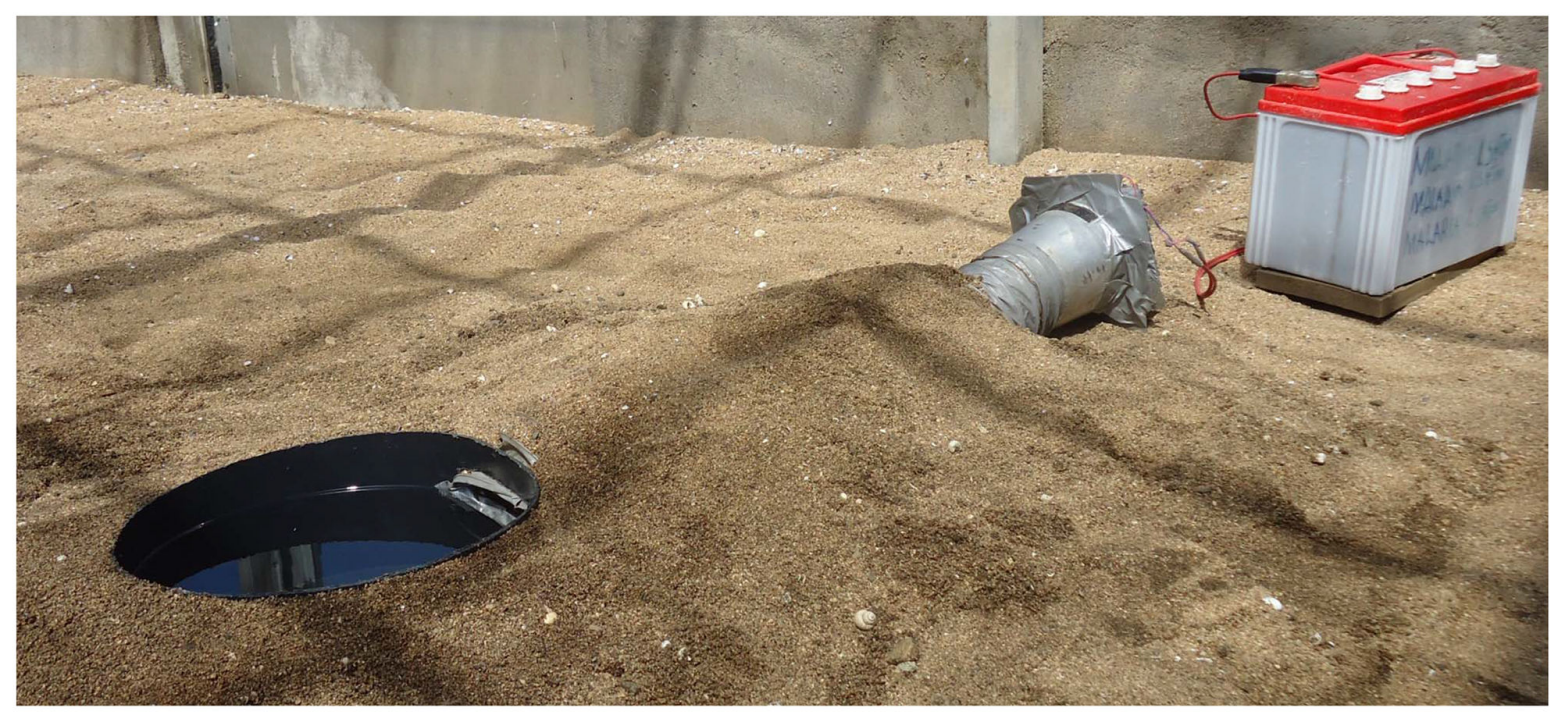
The OviART gravid trap set up. Trap was set by sinking the water-filled bucket into the ground so that the lip of the bucket was flush with the sand surface. The suction tube was buried in the sand leaving only the end with the fan exposed above the soil in order to let airflow freely.

The OviART gravid trap was set by sinking the water-filled bucket into the ground so that the lip of the bucket was flush with the sand surface. The suction tube was buried in the sand leaving only the end with the fan exposed above the soil in order to let airflow freely (Figure 5). Gravid mosquitoes passed through this tube into the collection chamber. The fan was powered by a 12 V 50 Ah lead acid battery (Chloride Exide Ltd, Kenya). The trapping efficacy of the OviART gravid trap was compared with the Box gravid trap in two choice bioassays. The number of mosquitoes caught in the traps and the number of eggs in the ponds was recorded.

In a final step a single OviART prototype trap was tested nightly for 12 nights in a semi-field system. The intention was to determine the proportion of released *An. gambiae s.s.* that could be collected by the trap. The trap was rotated randomly between all four sites in the semi-field system. The number of mosquitoes trapped was recorded. 

### Data analysis

The data were analysed using generalized linear mixed effects models. The analyses were done with R statistical software version 2.14.2 including the contributing packages MASS, lme4, glht and multcomp [50]. Previous experiments [43] have shown that mosquito responses are highly variable between different batches of mosquitoes and between different sites in the greenhouse irrespective of the test treatments. Therefore, the night of experiment (same batch of mosquitoes) and location (site) where the traps were placed in the semi-field system were included in the models as random factors. To adjust for excess variation between rows (data points) recording the number of trapped mosquitoes (overdispersion) a factor was created with a different level for each row of the data set and also included as a random factor in the model. The experimental treatments were entered as fixed effects. A Poisson distribution of the data with a log link function were used. All mean counts per treatment and their 95% confidence intervals (CIs) were calculated as the exponential of the parameter estimates for models with no intercept included [51]. Similarly, multiple comparisons of treatments were calculated based on the model parameter estimates.

## Results

### Trap comparison

**Figure 6:**
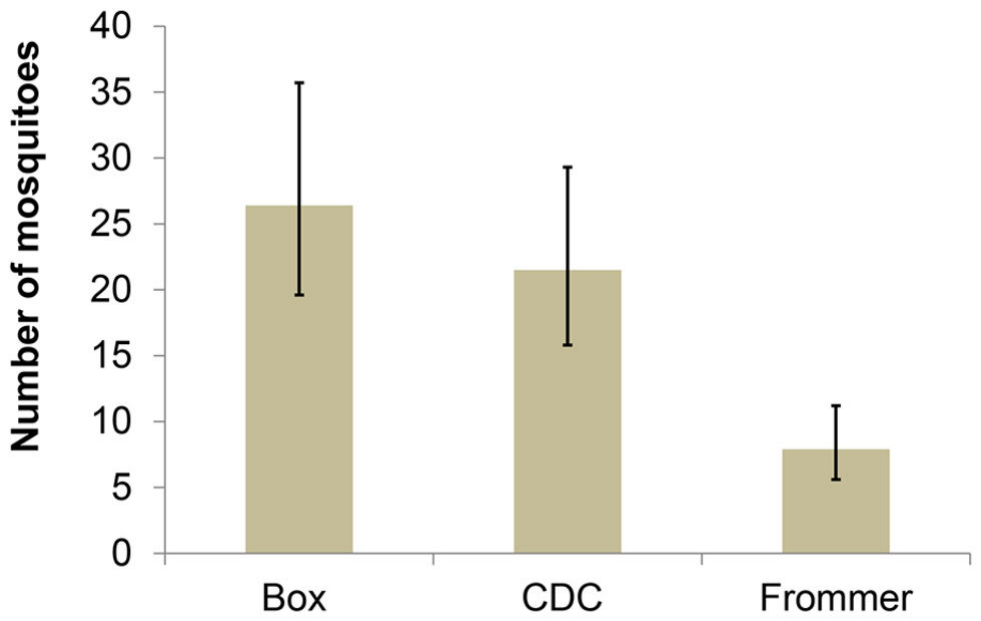
Mean *Anopheles gambiae s.s* catch sizes of CDC, Frommer updraft and Box gravid traps. Error bars equal the 95% confidence intervals; 200 gravid females were released in semi-field system per night for 12 nights.

*An. gambiae s.s.* females were caught in all three traps in the semi-field system (Figure 6), but the total mean number trapped per night was low (59.3, 95% CI 50.3–70.0) i.e. <30% of released mosquitoes were recovered by the three traps. The Box gravid trap and the CDC gravid trap collected similar numbers of mosquitoes (RR 0.8, 95% CI 0.6–1.2; p= 0.284). In contrast, it was 70% less likely to collect a mosquito with the Frommer updraft gravid trap (RR 0.3, 95% CI 0.2–0.5; p < 0.001) compared with the other two traps (Figure 6). On average, 858 (95% CI 570-1291) eggs were collected from the open pond per day, indicating that the low catch numbers in the traps were not because released mosquitoes did generally not respond due to environmental factors or because they were not gravid. Rather it appears that the females preferred to approach the open pond than a pond with a trap on top. Only few and similar numbers of eggs were found in ponds with traps (average for all three traps combined 125.1 (95% CI 82.8–189.0). This implies that most females that approached the pond were sucked into the trap before getting an opportunity to lay eggs, so low catch sizes are probably not due to weak suction of the fans.

### Trapping efficacy of the Box gravid trap

Based on the trap comparisons the Box gravid trap was selected for further evaluation in the following series of experiments since it caught the greatest number of mosquitoes and provided protection for battery, cables and mosquitoes which would be an added advantage when used in the field during wet weather [38,40].

The number of mosquitoes approaching the Box gravid trap was reduced by 30% compared to the number that approached the open pond, irrespective of whether the trap was switched on, creating a distinct sound, or switched off and silent (Table 1). This suggested that the presence of the Box on top of the pond deterred mosquitoes from approaching the site and led to the next experiment. 

**Table 1:** Results of the statistical analyses of the individual experiments implemented to develop a new gravid trap for *Anopheles gambiae s*.*s*.

**Treatment**	Mean (95% CI)*	**RR (95% CI)**		**p**
**Response of gravid *An.gambiaes.s.* to a pond with an operating Box gravid trap**				
Pond only	62.1 (40.2–95.8)		1	
Trap over pond	40.1 (25.9–62.1)	0.7 (0.6–0.7)		< 0.001
**Response of gravid *An.gambiaes.s.* to a pond with a soundless Box gravid trap**				
Pond only	51.2 (39.6–66.1)		1	
Soundless trap over pond	36.9 (28.4–47.9)	0.7 (0.6–0.8)		<0.001
**Response of gravid *An.gambiaes.s.* to a pond with a Box gravid trap next to it**				
Pond only	32.5 (22.8–46.5)		1	
Trap next to pond	33.4 (23.4–47.7)	1.0 (0.9–1.2)		0.693
**Comparison of trapping efficacy of the prototype OviART gravid trap and the Box gravid trap**				
Box gravid trap	25.2 (19.1–33.3)		1	-
OviART gravid trap	41.3 (31.6–53.9)	1.6 (1.2–2.2)		0.001

CI = confidence interval RR = relative rate * predicted by using the parameter estimates of the mixed effects model.

### Development of a new gravid trap

A test was designed where the number of mosquitoes that visited a pond with a Box gravid trap set next to it was compared with a pond without a trap. Similar numbers of females visited the two ponds (Table 1).

Based on the analyses of factors that affected the approach of gravid *An. gambiae s.s.* to a Box gravid trap a prototype of a new gravid trap (OviART gravid trap) was developed. Here the catching device was moved to the side of the pond; 60% more *An. gambiae s.s.* were collected by the OviART gravid trap prototype than the Box gravid trap (Table 1). A large difference was found in the egg numbers recovered from the ponds of the two traps. Eggs were only found on three out of the 12 collection nights in the pond of the new OviART trap (in total 87 eggs). In contrast, eggs were found nightly and nearly 19 times more eggs (total 1652) were laid in the pond of the Box gravid trap over the 12 nights period. When evaluated alone, the OviART prototype recollected approximately one third of the released mosquitoes in the single choice bioassay (31.9%, 95% CI 20.4-46.4%).

## Discussion

In the situation where gravid *An. gambiae s.s.* females had a choice to oviposit in an open pond or in ponds with a trap on top <30% of the released mosquitoes were collected by the three commercially available gravid traps in the semi-field system. The Box and CDC gravid traps showed similar efficacy whilst the Frommer updraft gravid trap trapped relatively few mosquitoes. The extremely low efficacy of the Frommer updraft gravid trap may be due to its physical features since the base of this trap stands inside the water-filled basin. To be trapped, mosquitoes have to fly under the base of the trap that is only about 3 cm above the water surface. The lower volume of water as compared to the other traps might have also contributed to the lower catch size due to a lower release of water vapour. The low efficacy of this trap is consistent with recent observations by Irish et al. [47] for *Culex* *quinquefasciatus* .

It is interesting to note that the overall catching efficacy of the CDC and Box gravid trap under semi-field conditions falls into the same range as reported for gravid culicine mosquitoes where recollections were highly variable and between 22% and 63% of the released mosquitoes [38,52,53]. However, it is difficult to make direct comparisons between the efficacy of these traps for culicine and anopheline mosquitoes since (1) our semi-field system had a much greater volume than those used for culicines [38,40], (2) unlike the experiments for culicines which use attractive infusions and semiochemicals there are no confirmed attractants for *An. gambiae* s.s. available and (3) in our trap comparison the open pond competed with the traps and therefore might have diverted a proportion of the mosquitoes and reduced the number of mosquitoes approaching each trap.

There are very few reports of the trapping efficacy of commercially available gravid traps for collection of *An. gambiae* s.l., or any other anopheline species in the field and surprisingly none of these were set intending to collect malaria vectors. Nevertheless, there is a notion that these traps might be less suitable for anophelines than for culicines based on the actual trapping results [47,54]. This has to be cautiously interpreted because most gravid traps are used to collect culicine females and were baited with a range of fermented plant infusions which might repel malaria vectors which are generally associated with less strongly polluted water [55–57]. The only indication that it might be possible to sample malaria vectors with commercially available gravid traps comes from the work of Muturi and colleagues [54]. In outdoor collections in a rice agro-ecosystem they collected approximately 5-6 gravid anophelines each night with a grass-infusion baited CDC gravid trap compared with 18-20 host-seeking anophelines in a CO_2_-baited CDC light trap [54]. Since host-seeking collections are usually higher than others [19], this ratio is encouraging for the development of a gravid trap for malaria vectors. Fresh water instead of grass-infusion might have increased the trapping result for anophelines in this study.

In our study the Box gravid trap was selected for further evaluation since its compact design meant that the internal parts were well protected from the elements, which would be an advantage during the rainy seasons, but it was found that the approach of gravid females was significantly reduced when the Box trap was positioned directly over a pond, compared to a pond alone. This effect was not due to the sound of the trap and the removal of the trapping device off the pond confirmed that the females were visually deterred by the presence of the trapping device directly on the oviposition medium.

Previous work has shown that *An. gambiae* mosquitoes first evaluate a potential larval habitat before making a decision to lay eggs [57–62]. The decision to lay eggs might be based on visual or chemical cues or a combination of both [63]. Mosquitoes in flight depend on optical inputs to orient themselves, identify and access a target [64–67]. Visual cues are believed to be long range cues important for gravid mosquitoes to identify different habitats and specific oviposition site characteristics before they evaluate the habitat using chemical signals received by olfactory receptors, hygroreceptors and contact chemoreceptors [63,68–71]. The visual parameters include shape, size, contrast, light quality and intensity, texture and colour of a pond [58,62,63,72–76]. *An. gambiae s.s.* prefers open sunlit habitats [59,77–79] and avoids habitats densely covered by vegetation that create obstacles to oviposition [55,62,80]. A recent study suggested that shiny sticky film attracted *An. gambiae s.s.* due to its close resemblance to water [43]. This phenomenon was also exploited by Harris and colleagues who developed a simple collection device for gravid mosquitoes using a transparent acetate sheet coated with insect glue placed to float on the edge of natural aquatic habitats [36].

The most likely reasons that the Box trap does not attract many gravid females are because the pond is too shady and the large trap over the water surface impedes their pre-oviposition flight. The new OviART gravid trap provided female *An. gambiae s.s.* with an open oviposition site which improved the catch size by 60% compared to the Box gravid trap. When the OviART trap was evaluated alone, approximately one third of all released mosquitoes were trapped. This corresponds well with observations we made previously when studying (1) gravid females’ approach towards an artificial pond surrounded by complete square of electrocuting nets and (2) the numbers of mosquitoes drowned in an artificial pond treated with detergent [43]. The absence of eggs in the pond of the new trap during most of the nights also indicates that the great majority of the females that approach the ponds with the intention to lay eggs got sucked into the collection chamber.

The disadvantage of the OviART prototype trap is that the collection device and the battery are less protected from the elements compared to the Box gravid trap. Nevertheless, modifications might be possible to improve the design by providing a casing for both the collection device and battery. Furthermore, to power the stronger fan needed to suck mosquitoes from the entire water surface a larger battery was required that makes the trap more difficult to transport and increases the risk of theft when used outdoors. The strong suction in the trap, forcing mosquitoes into the netting at speed, probably contributed to some of the mosquitoes found dead in the trap. Since collection of undamaged gravid females is advantageous for the isolation of a number pathogens (other than malaria parasites that can be identified from dry specimen) [38,40,48,49] future modifications should aim to improve the survival of mosquitoes in the trap. Future work should also evaluate the airflow of the trap and its impact on attracting mosquitoes. Sucking the air from above the water surface through the collapsible pipe channels potential volatile chemicals from the water surface to the side of the pond, which might affect the response towards the trap especially if attractant semiochemicals were used [47,81]. To increase user safety and longevity a gel battery should be considered in future instead of a lead acid battery. Whilst the required battery is expensive (approximately $100–120) all the other parts can be made from locally available plastic ware and electrical supplies. Costs for the entire trap are estimated to be less than $150, which is still cheaper than the commercially available Box gravid trap ($192) [82] but significantly more expensive than simple catching devices as the sticky transparency [36] or simply a pond treated with detergent which has shown to work well under semi-field conditions [43]. The advantages of the OviART gravid trap are: (1) that gravid females are caught alive which eases species identification and provides potential for pathogen identification other than malaria; (2) that the trap does not depend on the presence of natural habitats but can be moved around and located close to human habitation or close to aquatic habitats depending on the research question, and (3) that environmental conditions (e.g. rain, dust, predation) are less prone to affect the catch size and quality of specimen than with sticky material and detergents. The trap may be further improved should attractive semiochemicals be discovered for *Anopheles gambiae* s.l. that could be added to the water increasing the traps competitiveness with natural habitats.

## Conclusion

The three commercially available gravid traps tested in this study were specifically developed for collecting culicine mosquitoes that differ greatly in their oviposition behaviour from the malaria vector *An. gambiae* s.s. [59,83]. Nevertheless, the Box and CDC gravid trap caught consistent numbers of this species under semi-field conditions but their performance was not considered satisfactory enough to evaluate them under field conditions. The present work revealed that gravid *An. gambiae* females were visually deterred by the presence of the trapping device directly on the oviposition medium. Based on these investigations, a gravid trap was developed that provides open landing space for egg-laying female mosquitoes which improved the catch size by 60% compared to the Box gravid trap. The efficacy of this prototype trap under semi-field conditions is promising and warrants further investigations to further improve the catch size by modifying the fan suction, the size of the oviposition bowl, and physical characteristics of the trap (e.g. include visual contrast), and to improve the physical structure of the trap and its components to reduce costs and increase durability.

Field evaluations under different ecological conditions have to be carried out to confirm the trapping efficacy since semi-field systems are limited by their size, which does not allow us to study the natural completion of long-range kinesis. Furthermore, field tests are needed to assess how competitive the OviART gravid trap will be in the field especially in areas with extensive aquatic habitats and during rainy seasons. Nevertheless, previous studies have shown that similar artificial habitats made of plastic tubs buried into the ground are well accepted by anophelines in areas with high habitat density and get colonised rapidly and densely during dry and rainy seasons [84–86]. An effective gravid trap for malaria vectors will not only be useful for monitoring population densities but also enables studies of vector dispersal in search of aquatic habitats, an area of research that has received very little attention to date [87,88].
